# Impact of Maternal Smoking on the Onset of Breastfeeding versus Formula Feeding: A Cross-Sectional Study

**DOI:** 10.3390/ijerph16244888

**Published:** 2019-12-04

**Authors:** Carolina Lechosa Muñiz, María Paz-Zulueta, Elsa Cornejo del Río, Sonia Mateo Sota, María Sáez de Adana, María Madrazo Pérez, María Jesús Cabero Pérez

**Affiliations:** 1 Hospital Universitario Marqués de Valdecilla, 39008 Santander, Spain; carolina.lechosa@scsalud.es; 2 Faculty of Nursing, Universidad de Cantabria, IDIVAL, GI Derecho Sanitario y Bioética, GRIDES, 39008 Santander, Spain; 3 Obstetrics Service, Hospital Universitario Marqués de Valdecilla, 39008 Santander, Spain; elsacdelrio@gmail.com (E.C.d.R.); soniamsota@yahoo.es (S.M.S.); 4 Gynecology Service, Hospital Universitario Marqués de Valdecilla, 39008 Santander, Spain; maria.saezdeadana@scsalud.es; 5 Faculty of Nursing, University of Cantabria, 39008 Santander, Spain; maria.madrazo@unican.es; 6 Pediatrics Service, Hospital Universitario Marqués de Valdecilla, 39008 Santander, Spain; mariajesuscabero@gmail.com

**Keywords:** breastfeeding, newborn, prevalence, tobacco

## Abstract

This study sought to estimate the prevalence of maternal smoking and its association with exclusive breastfeeding vs. formula feeding. A cross-sectional study was performed on postpartum women at a public hospital in Spain, between January and August 2018. The main variables studied were their age, level of study, smoking habits, and chosen mode of infant feeding. In total, 948 postpartum women were included. Of these, 12.45% were smokers who smoked a mean of 7.23 cigarettes/day. Among the group of smokers, the probability of feeding the newborns with formula milk was multiplied by 2.32 ([95%CI 1.50–3.58] *p* < 0.001). When stratifying tobacco use into mild, moderate and severe, we found a statistically significant dose–response pattern. These associations and their statistical significance were maintained when adjusting by age and level of study. In conclusion, in the group of postpartum mothers who smoked, the probability of feeding the newborns with formula milk doubled. Our data highlight the need to improve health education programs in women of childbearing age, especially during pregnancy.

## 1. Introduction

The World Health Organization (WHO), the American Academy of Pediatrics (AAP), and the Spanish Association of Pediatrics (AEP) recommend exclusive breastfeeding for up to 6 months, and then continued breastfeeding combined with solid foods for 2 years or more, for as long as the mother and baby desire [1,2]. 

In Spain, according to the National Health Survey of 2017 [3], 74% of mothers continue to breastfeed at 6 weeks. An important decrease subsequently occurs at 6 months, when only 39% of mothers breastfeed. These data demonstrate that the current national rates of breastfeeding continue to be below the recommendations of both national and international organizations [4,5,6].

In Cantabria, the results of the Cantabrian Health Survey of 2006 (ESCAN-06) [7] reveal that maternal breastfeeding progressively decreases from birth until 6 months, at which point infants are provided formula milk. Thus, 66.4% of mothers maintain exclusive breastfeeding until 6 weeks after birth, which then decreases to 45.9% at 3 months, and 19.4% at 6 months.

Several studies have found that certain socio–demographic characteristics such as age, parity, level of study, socioeconomic level, and smoking habits can influence the initiation and duration of exclusive breastfeeding [8,9].

The aim of our study was to estimate the prevalence of maternal smoking and its association with the onset of breastfeeding vs. formula feeding, at a hospital of reference within the community. 

## 2. Materials and Methods 

A cross-sectional study was performed. Based on the study’s aims, the required sample size was 805 newborns, therefore, 966 total newborns were recruited, considering the potential attrition or loss of 20% of the sample. The sample included all the newborns consecutively born until the necessary sample was obtained. The study period was from 1 January 2018 to 31 August 2018. 

### 2.1. Data Sources

The data analyzed in this study were obtained based on the clinical records of both the newborn and the mother. The computer applications, Gacela Care and Visor Corporativo, were used to compare or complete the information in cases where the record did not appear in the register. The collected data comprised the age of the mother, their smoking tendency, their level of study, their maternal work status, how many weeks of pregnancy, the newborn weight and the feeding method. 

The smoking tendency was classified based on the classification by the WHO, and grouped according to the number of cigarettes smoked, into mild smokers (less than 5 cigarettes per day), moderate smokers (an average of 6 to 15 cigarettes per day), and severe smokers (more than 16 cigarettes per day on average). 

Regarding the maternal level of study, this was categorized into primary studies, high school, vocational training, university studies, and postgraduate studies. Work status was categorized into employed, unemployed, inactive or student.

The week of pregnancy variable was categorized into newborn, post-term, full-term, late preterm, very preterm, or extremely preterm [10]. The newborn feeding method was classified as either exclusive breastfeeding (the newborn only receives breast milk), mixed feeding (the newborn receives breastmilk and formula feeding), formula feeding (the newborn has formula milk), or donated milk (the newborn is fed donated human milk).

### 2.2. Statistical Analysis

An initial descriptive analysis was incorporated. For the categorical and discrete variables, proportions were estimated by their corresponding 95% confidence intervals (95% CI), using Pearson’s chi-squared test for comparisons, or, alternatively, using Fisher’s exact test when over 20% of the fields presented a number of expected cases that were less than or equal to 5. For the continuous variables, the mean and standard deviation (SD) were estimated. The Kolmogorov–Smirnov test was used to determine the normality of the distribution. Crude and adjusted odds ratios (OR) were estimated with their 95% CI using unconditional logistic regression. The alpha error was set at 0.05 and all p values were bilateral. All the statistical analyses were performed using the SPSS v22.0 (IBM SPSS Statistics for Windows. Armonk, NY: IBM Corp.).

### 2.3. Ethical Considerations

This study was approved by the Research Ethics Committee of Cantabria on 21 July, 2017. During the hospital stay after birth, patients were informed of the existence of the study and they were requested to sign the informed consent form to participate in the study.

## 3. Results

In total, 970 newborns were included. Of these, 50.52% were male and 49.49% were female. The mean gestational age at the time of birth was 39.09 ± 1.96. Up to 93.81% were full-term births, 4.02% were late preterm births and 2.17% were preterm births. The mean weight at birth was 3244.55 ± 572.33 g, ranging from 870 to 4840 g. The prevalence of exclusive breastfeeding at hospital discharge was 53.40% (Table 1).

The mean age of postpartum women was 33.7 ± 5.23, ranging from 17 to 52 years. Our results revealed statistically significant differences in smoking habits and level of study, when stratified by newborn feeding methods. The remaining sample characteristics are detailed in Table 2 and Table 3. 

When analyzing smoking tendency, we found that 12.5% of postpartum women (*n* = 118) were smokers, with a mean consumption of 7.2 cigarettes/day. 

In the group of smoking women, the probability of feeding newborns with formula milk was multiplied by 2.32 ([95% CI, 1.50–3.58] *p* < 0.001). When stratified by tobacco smoking in mild, moderate and severe, a statistically significant dose–response pattern was found. With a consumption of ≥6 cigarettes/day, the probability of feeding the newborns with formula milk was multiplied by 3.09 ([95% CI 1.75–5.45]), compared to 1.72 ([95%CI 0.94–3.18]) in the group of mild consumers. These associations and the statistical significance were maintained when adjusting by age and level of study at 3.09 [95% CI 1.75–5.45] *p* < 0.001 (Table 4). 

## 4. Discussion

The rate of exclusive breastfeeding in full-term infants at hospital discharge was 54.95%, which is a lower average than in other communities [3,11,12,13,14,15]. This continues to be far from the rates required by the Initiative for the Humanization of Assistance to Birth and Lactation (IHAN) initiative, which establishes that at least 75% of infants should be exclusively breastfed from birth until discharge. 

In our study, we found that most mothers opted for breastfeeding, with formula feeding rates of 17.94%. These data are similar to those reported in other parts of Spain. Without addressing gestational age, the prevalence of exclusive maternal breastfeeding at hospital discharge was 53.40%, 28.04% received mixed feeding, and 0.6% were using donated human milk, as our community had a milk bank [16,17]. 

Bearing in mind the low rates of maternal breastfeeding in our hospital, which are well below those recommended by the WHO, one of the objectives of the Cantabrian Health Service since 2017 has been to improve the practices that promote and protect breastfeeding. Thus, the hospital is immersed in the process of Baby-Friendly Hospital Initiative (BFHI) accreditation, now called the Initiative for the Humanization of Assistance to Birth and Lactation.

The IHAN accreditation is targeted at health professionals working at hospitals, by providing an effective, evidence-based accreditation program, structured and externally evaluated, which enables and facilitates the work of initiating, supporting and increasing the duration of breastfeeding. The IHAN accreditation is a designation which is granted to hospitals that fulfill the following requisites:

1. Fulfill the Ten Steps to a Successful Breastfeeding: based on scientific evidence and approved by national and international agencies as standards of good clinical practice.
2. Implement the Code of Marketing of Breast-milk Substitutes and the related, later resolutions by the World Health Assembly.
3. Embrace at least a 75% rate of exclusive breastfeeding (from birth until hospital discharge).
4. Furthermore, the mother–child bond must be promoted, regardless of whether or not the mother decides to breastfeed, providing truthful information and support, and instructing them in the preparation and safe administration of bottles of breast milk substitutes.
5. Likewise, a hospital that is accredited is compelled to perform assistance to birth according to the “Strategy of Care for Normal Birth of the National Health System”. Therefore, when the clinical situation of the mother and child allows for it, their needs will be respected, the creation of a mother–child bond will be promoted, and the early initiation of breastfeeding will be supported.

The process of obtaining IHAN accreditation in a maternity ward is highly complex, as it entails a significant effort on behalf of the professionals involved and the managers of the institution. To facilitate this, the IHAN suggests a gradual implementation in four phases: Phase 1D (Discovery), Phase 2D (Development), Phase 3D (Dissemination), and Phase 4D (Designation).

On this path towards recovery, studies such as this are highly relevant, in that they provide objective data on the influence of smoking on the initiation of breastfeeding. This paper reveals how a modifiable factor, such as smoking, significantly influences the decision to breastfeed. 

Numerous factors have been reported to influence maternal breastfeeding [14,15,18,19,20,21,22,23]. In our study, we confirmed the significance of the maternal educational level, weeks of pregnancy and smoking tendency. 

The prevalence of smoking in the population of pregnant women was 12.5%, with a mean of 7.2 cigarettes/day, which is similar to the number obtained in other communities [24], and very similar to the general population of Spanish women who smoke (18.8%). These data are sufficiently striking, as with them we can say that 70% of female smokers continue smoking during their pregnancy. Besides this, we would have to add that the exact prevalence of smoking during pregnancy is hard to identify, because most of the data available are based on declared, self-reported consumption. The studies which use biochemical markers have demonstrated that this prevalence of smoking is greater during pregnancy [25,26].

The mother’s decision to breastfeed her newborn may be influenced by maternal smoking habits, which is in line with previous studies performed in Spain [27,28], Europe [29,30,31,32] and internationally. In the latest published meta-analysis, maternal smoking habits are considered a high-impact factor on breastfeeding no smoking vs. smoking (RR = 1.76 [IC 95% 1.59–1.95]) [33]. In the group of women smokers, the probability of feeding the newborns with formula milk was multiplied by 2.32 ([95%CI 1.50–3.58] *p* < 0.001). When stratified by smoking consumption into mild, moderate and severe, we found a statistically significant dose–response pattern. Smoking during pregnancy constitutes one of the modifiable risk factors associated with adverse maternal, fetal and neonatal effects [34,35,36,37,38,39,40,41]

In Spain, the Spanish Society of Family and Community Medicine has drafted guidelines for the treatment of active and passive smoking. These guidelines are a reference on how to intervene in the case of patients who smoke and are attending primary care consultations [42], and may be useful for implementing health education programs insisting on smoking cessation for fertile women, as well as pregnant. 

### Limitations

In studies based on secondary information (records), one of the main limitations is the low quality of the information. This low quality could be due to inconsistencies in the information provided in different records, or to the insufficient completion of the medical records required for the study. To minimize these biases, prior to the study, we selected the variables which are more homogenously, systematically and objectively gathered in electronic clinical records. Likewise, prior to the definitive inclusion of the variables, the concordance among the data of the different sources used was assessed.

The observational design of this study does not enable the establishment of a causal relationship between variables. However, we can characterize the frequency and/or distribution of the study phenomenon based on the study variables.

Our tobacco consumption data come only from self-reported consumption. Nowadays, we know that it could be made more accurate by using biomarkers, but we were unable to perform a homogeneous and systematic analysis of biomarkers (for example, to conduct an analysis of nicotine/coinine in cord blood). For this reason, such data could not be included in the analyses.

Nonetheless, we have used the same methods as most of the studies published on the rates and prevalence of smoking during pregnancy, which have been performed with data of declared or self-reported consumption [42,43,44,45,46].

## 5. Conclusions

In the group of postpartum mothers who smoked, the probability of feeding the newborns with formula milk doubled. Our data highlight the need to improve health education programs in women of childbearing age, especially during pregnancy. All women of reproductive age who smoke and who hope to have offspring should be offered a health program directed at smoking cessation. This would avoid adverse maternal, fetal and neonatal effects and improve breastfeeding rates.

## Figures and Tables

**Table 1:** Descriptive analysis of newborns included during the study period (2018).

Variable	*n* = 970	%	CI95%CI		Range
Gender					
male	490	50.52	47.32	53.71	
female	480	49.49	46.29	52.68	
Weeks of pregnancy: mean [SD]	39.09	1.96			range 25–42
Full-term	910	93.81	92.25	95.38	
Late preterm	39	4.02	2.73	5.31	
Preterm	21	2.17	1.20	3.13	
Weight: mean [SD]	3244.46	572.33			range 870–4840
Normal weight	806	83.09	80.68	85.50	
Macrosomia	80	8.25	6.47	10.03	
Low birth weight	84	8.66	6.84	10.48	
Type of feeding					
Exclusive breastfeeding	518	53.40	50.21	56.59	
Mixed feeding	272	28.04	25.16	30.92	
Formula feeding	174	17.94	15.47	20.40	
Donated human milk	6	0.62	0.07	1.16	

**Table 2:** Descriptive analysis of each of the postpartum women included during the study period (2018).

	*n* = 948	% *	95%CI		Range
Maternal age: mean [SD]	33.68	5.23			range 17–52
Twins					
no	926	97.68	96.67	98.69	
yes	22	2.32	1.31	3.33	
Smoking					
no	830	87.55	85.40	89.71	
yes	118	12.45	10.29	14.60	
Number of cigarettes: mean [SD]	7.23	5.25			
Maternal educational level					
Did not complete high-school	214	22.57	19.86	25.29	
High school	111	11.71	9.61	13.81	
Vocational training	273	28.80	25.86	31.73	
University studies	343	36.18	33.07	39.29	
Post-graduate education	7	0.74	0.14	1.34	
Work situation					
Employed	660	69.62	66.64	72.60	
Unemployed	162	17.09	14.64	19.54	
Inactive	116	12.24	10.10	14.38	
Student	10	1.06	0.35	1.76	
Employment					
Inactive/unemployed	275	29.01	26.07	31.95	
Directors and managers	7	0.74	0.14	1.34	
Technicians/PCSE	161	16.98	14.54	19.43	
Other technicians/intellectuals	58	6.12	4.54	7.70	
Technicians/Support professionals	47	4.96	3.52	6.39	
Office without public	62	6.54	4.91	8.17	
Office with public	29	3.06	1.91	4.21	
Catering and retail	142	14.98	12.65	17.30	
Health and care	90	9.49	7.58	11.41	
Safety	1	0.11	0.00	0.59	
Manufacturers and assemblers	19	2.00	1.06	2.95	
Services sector (not transport)	53	5.59	4.08	7.11	
Laborers and transport	4	0.42	0.12	1.08	

* Valid percentage (without considering the missing or unknown data).

**Table 3:** Descriptive analysis of postpartum women during the study period (2018) considering the feeding method.

	Feeding Method upon Hospital Discharge																
	EB *				Mixed				FF **				DHM ***				
	*n* = 516	%	95%CI		*n* = 262	%	95%CI		*n* = 168	%	95%CI		*n* = 4	%	95%CI		*p*
Maternal age: mean [SD]	33.68 [5.02]				34.04 [5.32]				33.00 [5.54]				36.50 [11.68]				*0.156*
Parity																	
Primiparous	257	27.05	24.18	29.30	164	17.26	14.81	19.72	82	8.63	6.79	10.47	2	50	6.76	93.24	
Multiparous	259	27.26	24.38	30.15	98	10.32	8.33	10.30	86	9.05	7.18	10.93	2	50	0.76	93.24	*<0.001*
Smoking																	
no	465	90.12	87.44	92.79	231	88.17	84.07	92.27	131	77.98	71.41	84.54	4	100	39.76	100	
yes	51	9.88	7.21	12.56	31	11.83	7.73	15.93	37	22.02	15.46	28.59	0	0	0	60.24	*0.015*
Nº of cigarettes: mean [SD]	6.61 [5.14]				5.87 [3.32]				9.28 [6.20]								
*p*	*0.015*				*0.018*				*0.018*								
Educational level																	
Primary studies	91	17.64	14.25	21.02	67	25.57	20.10	31.05	56	33.33	25.91	40.76	0	0	0	60.24	
High school	59	11.43	8.59	14.28	31	11.83	7.73	15.93	21	12.5	7.201	17.8	0	0	0	60.24	
Vocational training	148	28.68	24.68	32.68	69	26.34	20.81	31.86	53	31.55	24.22	38.87	4	100	39.76	100	
University studies	214	41.47	37.13	45.82	93	35.5	29.51	41.48	37	22.02	15.46	28.59	0	0	0	60.24	
Postgraduate	4	0.78	0.21	1.973	2	0.76	0.09	2.73	1	0.60	0.02	3.27	0	0.00	0	60.24	*<0.001*
Work status																	
Employed	366	70.93	66.92	74.95	180	68.70	62.90	74.51	113	67.26	59.87	74.66	3	75.00	19.41	99.37	
Unemployed	84	16.28	13.00	19.56	46	17.56	12.76	22.36	31	18.45	12.29	24.62	1	25.00	0.63	80.59	
Inactive	61	11.82	8.94	14.7	32	12.21	8.06	16.37	23	13.69	8.195	19.19	0	0	0	60.24	
Student	5	0.97	0.32	2.247	4	1.53	0.42	3.86	1	0.60	0.02	3.27	0	0.00	0	60.24	*0.975*

* Exclusive breastfeeding; ** Formula feeding; *** Donated human milk.

**Table 4:** Relationship between maternal smoking and breastfeeding during the study period (2018).

*n* = 948	Formula Feeding																	
	n		ORc	CI	95%	*p*	ORa1	CI	95%	*p*	ORa2	CI	95%	*p*	ORa3	CI	95%	*p*
Smoking	no	yes																
no	698	132	1				1				1				1			
yes	82	36	2.32	1.50	3.58	*<0.001*	2.23	1.44	3.45	*<0.001*	1.95	1.25	3.04	*0.003*	1.94	1.24	3.03	*0.004*
mild	46	15	1.72	0.94	3.18		1.64	0.88	3.04		1.51	0.81	2.80		1.49	0.80	2.78	
mod/severe *	36	21	3.09	1.75	5.45		2.98	1.68	5.28		2.49	1.39	4.45		2.48	1.38	4.43	
*p trend*			*<0.001*				*<0.001*				*0.001*				*0.001*			

ORa1: odds ratio adjusted by maternal age—continuous. ORa2: odds ratio adjusted by level of maternal studies. ORa3: odds ratio adjusted by maternal age—continuous, and level of maternal studies. * Moderate and severe consumption: mean consumption > 6 cigarettes/day.
